# Response to “Redundancy of terms is not an error but plays a positive role in composing search strategies”

**DOI:** 10.5195/jmla.2020.832

**Published:** 2020-01-01

**Authors:** Jose Antonio Salvador-Olivan, Gonzalo Marco-Cuenca, Rosario Arquero-Avilés

**Affiliations:** Professor, Department of Library and Information Science and Faculty of Medicine, University of Zaragoza, Zaragoza, Spain, jaso@unizar.es; Professor, Department of Library and Information Science and Faculty of Medicine, University of Zaragoza, Zaragoza, Spain, gmarco@unizar.es; Professor, Department of Library and Information Science, Complutense University of Madrid, Madrid, Spain, carquero@ucm.es

**To the editor,** we agree with Schoones [1] that redundant terms (and their morphological variants) are beneficial for search planning (i.e., search strategy composition), so it is advisable to consider all possible terms (and their morphological variants) that may be used to represent a concept.

We also agree that a search strategy is a “Livin’ Thing” and that its results dictate whether the initial search strategy is also the final strategy. However, the search strategy published in a systematic review must be the final strategy that was executed in the databases and that produced the results indicated in the flowchart. It is this search strategy that enables a study to be replicated and that will be evaluated in the peer-review process. In our study, we assessed the published strategies, not the initial or intermediate strategies that were tested.

Authors justify redundancy because the decision to include or exclude terms depends on the references retrieved, as the effect of the terms on the results is impossible to predict. In the proposed example, with the search statement of “massive chronic intervillositis”[tw] OR “chronic intervillositis”[tw] OR “chronic histiocytic intervillositis”[tw] OR “histiocytic intervillositis”[tw]

OR “intervillositis”[tw], it is known beforehand that “intervillositis”[tw] will retrieve all records that contain this term (individually or as part of a sentence), so other terms are unnecessary.

Redundant terms in a search strategy do not affect the retrieval of information. The principle of parsimony instructs us to eliminate that which is unnecessary. Applied to information retrieval, this principle prompts us to eliminate any terms or phrases from a search strategy that do not retrieve or provide new records, as they are thus unnecessary.

**José Antonio Salvador-Oliván, MD, PhD,** jaso@unizar.es, https://orcid.org/0000-0001-8568-3098, Professor, Department of Library and Information Science and Faculty of Medicine, University of Zaragoza, Zaragoza, Spain

**Gonzalo Marco-Cuenca**, gmarco@unizar.es, https://orcid.org/0000-0002-7149-6192, Professor, Department of Library and Information Science and Faculty of Medicine, University of Zaragoza, Zaragoza, Spain

**Rosario Arquero-Avilés,** carquero@ucm.es, https://orcid.org/0000-0002-3097-8734, Professor, Department of Library and Information Science, Complutense University of Madrid, Madrid, Spain
